# Identification of a Potentially Functional microRNA–mRNA Regulatory Network in Lung Adenocarcinoma Using a Bioinformatics Analysis

**DOI:** 10.3389/fcell.2021.641840

**Published:** 2021-02-18

**Authors:** Xiao-Jun Wang, Jing Gao, Zhuo Wang, Qin Yu

**Affiliations:** ^1^The First School of Clinical Medicine, Lanzhou University, Lanzhou, China; ^2^Department of Respiratory Medicine, Gansu Provincial Hospital, Lanzhou, China; ^3^Respiratory Medicine Unit, Department of Medicine, Karolinska Institute, Stockholm, Sweden; ^4^Department of Pulmonary Medicine, Helsinki University Hospital, University of Helsinki, Helsinki, Finland; ^5^Department of Pathology Medicine, Gansu Provincial Hospital, Lanzhou, China; ^6^Department of Respiratory Medicine, The First Hospital of Lanzhou University, Lanzhou, China

**Keywords:** lung adenocarcinoma, microRNAs, hub genes, bioinformatics, prognostic marker

## Abstract

**Background:**

Lung adenocarcinoma (LUAD) is a common lung cancer with a high mortality, for which microRNAs (miRNAs) play a vital role in its regulation. Multiple messenger RNAs (mRNAs) may be regulated by miRNAs, involved in LUAD tumorigenesis and progression. However, the miRNA–mRNA regulatory network involved in LUAD has not been fully elucidated.

**Methods:**

Differentially expressed miRNAs and mRNA were derived from the Cancer Genome Atlas (TCGA) dataset in tissue samples and from our microarray data in plasma (GSE151963). Then, common differentially expressed (Co-DE) miRNAs were obtained through intersected analyses between the above two datasets. An overlap was applied to confirm the Co-DEmRNAs identified both in targeted mRNAs and DEmRNAs in TCGA. A miRNA–mRNA regulatory network was constructed using Cytoscape. The top five miRNA were identified as hub miRNA by degrees in the network. The functions and signaling pathways associated with the hub miRNA-targeted genes were revealed through Gene Ontology (GO) analysis and the Kyoto Encyclopedia of Genes and Genomes (KEGG) pathway. The key mRNAs in the protein–protein interaction (PPI) network were identified using the STRING database and CytoHubba. Survival analyses were performed using Gene Expression Profiling Interactive Analysis (GEPIA).

**Results:**

The miRNA–mRNA regulatory network consists of 19 Co-DEmiRNAs and 760 Co-DEmRNAs. The five miRNAs (miR-539-5p, miR-656-3p, miR-2110, let-7b-5p, and miR-92b-3p) in the network were identified as hub miRNAs by degrees (>100). The 677 Co-DEmRNAs were targeted mRNAs from the five hub miRNAs, showing the roles in the functional analyses of the GO analysis and KEGG pathways (inclusion criteria: 836 and 48, respectively). The PPI network and Cytoscape analyses revealed that the top ten key mRNAs were NOTCH1, MMP2, IGF1, KDR, SPP1, FLT1, HGF, TEK, ANGPT1, and PDGFB. SPP1 and HGF emerged as hub genes through survival analysis. A high SPP1 expression indicated a poor survival, whereas HGF positively associated with survival outcomes in LUAD.

**Conclusion:**

This study investigated a miRNA–mRNA regulatory network associated with LUAD, exploring the hub miRNAs and potential functions of mRNA in the network. These findings contribute to identify new prognostic markers and therapeutic targets for LUAD patients in clinical settings.

## Introduction

Lung adenocarcinoma (LUAD) is the most common histological subtype of lung cancer, accounting for approximately 40% of all cases of lung cancer in China and other countries (Bray et al., 2018; Cao and Chen, 2019; Siegel et al., 2020). LUAD is characterized by rapid progression and early development of metastases and a high recurrence rate, with the highest morbidity and mortality in both genders (Bray et al., 2018; Cao and Chen, 2019; Siegel et al., 2020). The overall 5-year survival rates for LUAD only reach approximately 20% (Bray et al., 2018; Miller et al., 2019) given the lack of early detection and limited effective therapies at earlier stages of disease (Gan et al., 2017). LUAD patients have an 80% chance of surviving 5 years if diagnosed at an early stage (Henschke et al., 2006). Thus, there is a growing need to identify and characterize the molecular pathogenesis of LUAD in order to better understand the underlying disease mechanisms and to improve treatment outcomes of this malignancy.

MicroRNAs (miRNAs) are a family of small non-coding RNAs that downregulate gene expression by repressing or degrading messenger RNA (mRNA) targets, thereby controlling the genes involved in cellular processes (Di Leva et al., 2014; Du et al., 2018). miRNAs regulate the expression of approximately 30% of all human genes, playing important regulatory roles in many human diseases (Lewis et al., 2005; Di Leva et al., 2014; Ramassone et al., 2018; Wu K.L. et al., 2019; Wu S.G. et al., 2019; Condrat et al., 2020). miRNAs could be readily detected in circulation and carry information regarding the origin of a neoplasm, thus serving as diagnostic and prognostic biomarkers in the development of tumors (Hummel et al., 2010; Blondal et al., 2013; Qi et al., 2013; Zhang et al., 2016; Liu et al., 2017; Świtlik et al., 2019; Cojocneanu et al., 2020; Wang et al., 2020). An increasing number of studies have reported various expression levels of miRNAs in LUAD (Cazzoli et al., 2013; Zhong et al., 2018), although findings remain inconsistent. Extensive genomic studies have verified that abnormal expressions of multiple mRNAs of genes involved in LUAD tumorigenesis and progression play vital roles (Herbst et al., 2018). The miRNA–mRNA regulatory network is characterized in such a way that individual miRNA could regulate a wealth of different mRNAs of genes, and the individual mRNA of a target gene could be correspondingly suppressed by multiple different miRNAs (Cui et al., 2020; Li et al., 2020; Liu et al., 2020). Thus, it is necessary to examine the miRNA–mRNA regulatory network in LUAD to advance our understanding of its molecular mechanisms.

Microarray analysis has been widely used in the evaluation of miRNA in cancers to understand the complexity and heterogeneity of malignant disease (Jurj et al., 2017; Cojocneanu et al., 2020), but has not been fully elucidated in LUAD. A public dataset from the Cancer Genome Atlas (TCGA) is widely used in LUAD analyses, although cases primarily originate from a non-Asian population, yet include primary tissue samples. Hence, the TCGA dataset and actual clinical patient data from multiple sample types contribute to determining the biomarkers of LUAD and exploring the underlying mechanisms (Rheinbay et al., 2020).

Here, we constructed a miRNA–mRNA regulatory network of LUAD using TCGA data and our microarray analysis data, including multiple sample types and ethnicities, to subsequently identify hub miRNAs in the network. The functional enrichment analysis of the targeted mRNAs of hub miRNAs were investigated using the Gene Ontology (GO) and the Kyoto Encyclopedia of Genes and Genomes (KEGG) pathway enrichment analyses. In addition, we analyzed the protein–protein interaction (PPI) network to understand the potential mechanism of mRNAs in LUAD occurrence and development. In doing so, we identified the top ten key mRNAs using the PPI network and CytoHubba. The Gene Expression Profiling Interactive Analysis (GEPIA) allowed us to evaluate these key mRNAs and identify the hub genes in LUAD using survival analysis.

## Materials and Methods

**FIGURE 1:**
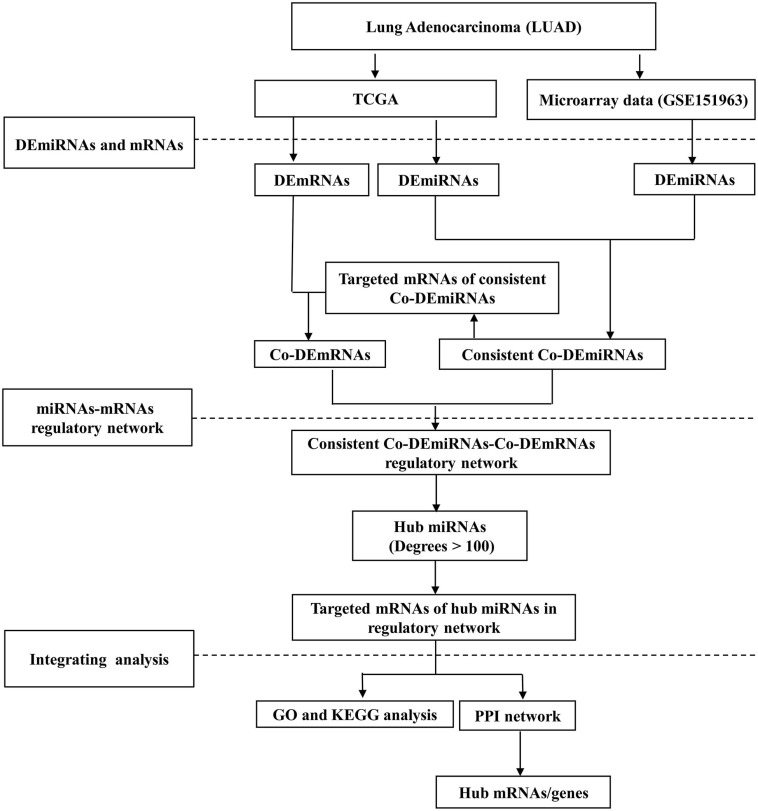
The study workflow. LUAD, lung adenocarcinoma; DE, differential expression; Co-DE, common differentially expressed; Co-mRNAs, common mRNAs; TCGA, the Cancer Genome Atlas; GO, gene ontology; KEGG, Kyoto Encyclopedia of Genes and Genomes; PPI, protein–protein interaction.

Figure 1 illustrates the workflow. This study received ethical approval from the Ethics Committee of the Gansu Provincial Hospital (14 April 2020, No. 2020-117). Informed consent was obtained from all participants in the microarray experiment, and the research adhered to the principles of the Declaration of Helsinki.

The TCGA dataset from tissue samples and the microarray data from plasma in LUAD were included in this study. Supplementary Tables 1,2 provide the clinical characteristics in LUAD from the TCGA and microarray data. The TCGA data included miRNA (400 LUAD and 15 normal lung tissue) and mRNA (515 LUAD and 20 normal lung tissue) samples, all downloaded using Firehose^1^ on 16 April 2020 (Izadi et al., 2017). The mRNA samples were obtained by filtering the samples with low-quality reads as expressions <0.8. The miRNA data were obtained from intersecting the mRNA-sequence data and filtering the samples with low-quality reads as expressions <0.5. The microarray data (*n* = 10) were collected between October 2018 and March 2019 at Gansu Provincial Hospital (China), and included initially diagnosed LUAD patients (*n* = 6) and controls (*n* = 4). We excluded patients who (a) previously received chemotherapy, radiotherapy, molecular-targeted therapy, immunotherapy or surgery before blood samples were collected; (b) had other combined cancers; (c) were pregnant or lactating; or (d) presented with cardiopulmonary insufficiency, serious cardiovascular disease and a serious infection as well as severe malnutrition (Wang et al., 2014; Li et al., 2019).

RNA degradation and contamination, especially DNA contamination, was monitored on 1.5% agarose gels. RNA concentration and purity were measured using the NanoDrop 2000 Spectrophotometer (Thermo Fisher Scientific, Wilmington, DE). RNA integrity was assessed using the RNANano 6000 Assay Kit of the Agilent Bioanalyzer 2100 System (Agilent Technologies, CA, United States). A total amount of 2.5 ng RNA per sample was used as the input material for the RNA sample preparations. Sequencing libraries were generated using NEBNext^*R*^ Ultra^TM^ small RNA Sample Library Prep Kit for Illumina^*R*^ (NEB, United States) following the manufacturer’s recommendations and index codes were added to attribute sequences to each sample. Raw data (raw reads) of fastq format were first processed through in-house perl scripts. The clean data (clean reads) were obtained by removing reads containing adapter, reads containing ploy-N and low-quality reads from raw data. The reads were trimmed and cleaned by removing sequences <15 nt or >35 nt. At the same time, Q20, Q30, and GC-content of the clean data were calculated. All downstream analyses were based on clean data with a high quality. Then, using the Bowtie soft tools, clean reads were compared to the Silva database, GtRNAdb database, Rfam database, and Repbase database, respectively, to identify the sequence alignment, and filtered for ribosomal RNA (rRNA), transfer RNA (tRNA), small nuclear RNA (snRNA), small nucleolar RNA (snoRNA), and other ncRNA and repeats. The remaining reads were used to detect known miRNA and novel miRNA predicted through comparison with known miRNAs from miRBase. Detailed information on the microarray appears elsewhere (Zhong et al., 2018). The sequencing reads were uploaded to the gene expression omnibus (GEO) dataset (access number GSE151963).

### Construction of the miRNA–mRNA Regulatory Network

The differentially expressed miRNAs (DEmiRNAs) and mRNA (DEmRNAs) were analyzed comparing the LUAD and normal samples using the R package *limma*. The DEmiRNAs consistent with the expression of TCGA were further analyzed. The isoform expressions of the DEmiRNAs were identified using the R package *miRNAmeConverter*. The common consistently and differentially expressed (Co-DE) miRNAs were obtained using intersected analyses comparing TCGA and microarray datasets (Cojocneanu et al., 2020). The targeted mRNAs of the Co-DEmiRNAs were predicted using three online analytical software tools including miRDB^2^, TargetScanHuman (version 7.2)^3^, and miRWalk (Agarwal et al., 2015; Dweep and Gretz, 2015; Chen and Wang, 2020)^4^. An overlap was applied to confirm the Co-DEmRNAs identified both in targeted mRNAs and DEmRNAs using FunRich tools (Pathan et al., 2015). Adjusted *p*-values (adj. p) were used to correct false-positive results (Patten et al., 2016). DEmiRNAs and DEmRNAs were identified applying *p* < 0.05 and | Log 2 fold-change (log2FC) | >1. The volcano plots were drawn using R package ggplot2. Based on the results of the Co-DEmiRNAs and Co-DEmRNAs, we constructed the miRNA–mRNA regulatory network using the Cytoscape software version 3.7.0 (Shannon et al., 2003). The top five miRNAs (degree >100) were selected as hub miRNAs in the network.

### Functional Enrichment Analysis and Confirmation of Hub Genes

The biological functions of the targeted mRNAs of these hub miRNAs were evaluated using the GO and KEGG pathway enrichment analyses with the R package ClusterProfiler (Yu et al., 2012). We considered *p* < 0.05 statistically significant. Using the STRING database,^5^ we downloaded data from the PPI network for targeted mRNAs for the hub miRNAs applying the following criteria: (a) homo sapiens; and (b) medium confidence 0.400 (Zhu et al., 2020). Then, the PPI network was further adjusted using the Cytoscape software version 3.7.0, and we identified the top ten key mRNAs of the PPI network using cytoHubba’s Maximal Clique Centrality (MCC) ranking (Yang et al., 2019). The hub mRNAs were further verified using survival analysis using the Gene Expression Profiling Interactive Analysis (GEPIA) tools^6^. Significant survival was identified through survival analysis (log-rank *p* < 0.05). The key mRNAs with significant survival outcomes were identified as hub genes in LUAD.

## Results

### Identification of Differentially Expressed miRNAs and mRNAs

**FIGURE 2:**
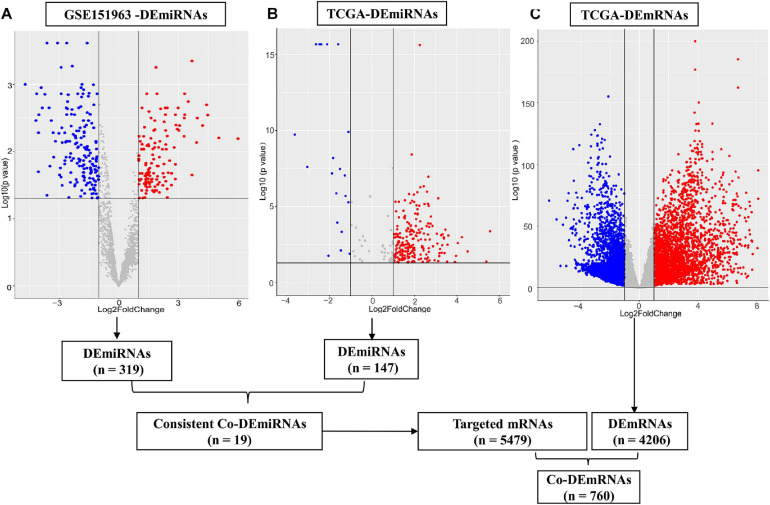
Differential expression of miRNAs and mRNAs. **(A)** Differential expression (DE) miRNAs in patient cohort (GSE151963), **(B)** DEmiRNAs in TCGA and **(C)** DEmRNAs in the TCGA dataset. The red plots show the upregulated expression of miRNA or mRNA; the blue plots show the downregulated expression of miRNA or mRNA; the gray plots show the normal expression of miRNA or mRNA. DE, differential expression; Co-DE, common differentially expressed; miRNA, microRNA; mRNA, messenger RNA.

Through small RNA sequence and data standardization, we harvested a total of 3,166 miRNAs for subsequent analysis of the microarray data. In total, we identified 6 patients (50% male, median age of 62.5 years, 100% Asian, and 100% stage III–IV) with LUAD using plasma samples from the microarray data. In total, 319 DEmiRNAs (145 upregulated and 174 downregulated miRNAs) were identified from the plasma comparing LUAD and controls in the microarray data (Figure 2A). In addition, we identified 515 LUAD patients (46% male, median age of 66 years, 2% Asian) from TCGA tissue samples, including stage I (53.4%), stage II (23.7%), and stage III–IV (25.2%). Furthermore, 4,206 DEmRNAs (1,105 upregulated and 3,101 downregulated) and 197 DEmiRNAs (126 upregulated and 71 downregulated) were identified using TCGA [(| log2 FC) | > 1 and *p* < 0.05, respectively, Figures 2B,C]. In addition, 147 of 197 DEmiRNAs (95 upregulated and 52 downregulated) exhibited isoform expressions.

### Constructing the miRNA–mRNA Regulatory Network

**FIGURE 3:**
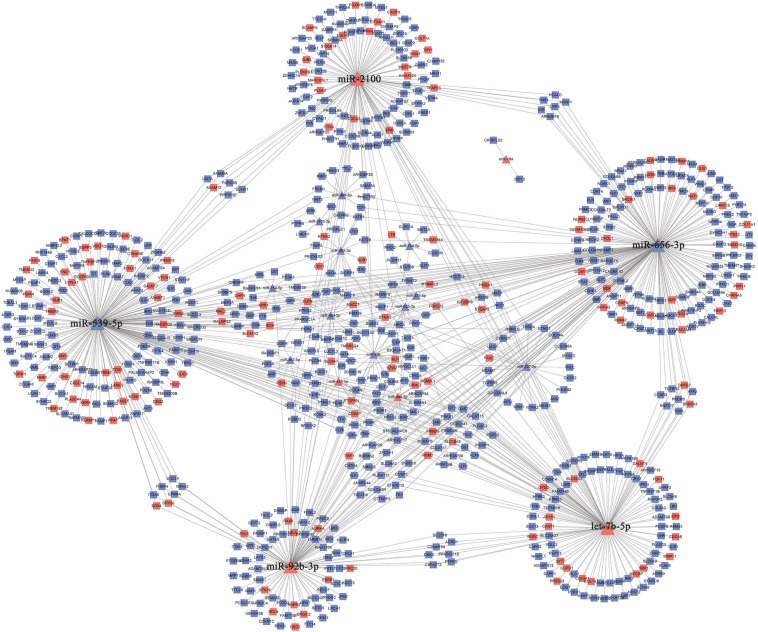
The miRNA–mRNA regulatory network. The rectangles and triangles represent mRNAs and miRNAs, respectively. Red represents the upregulated expression of miRNA or mRNA; blue represents the downregulated expression of miRNA or mRNA. miRNA, microRNA; mRNA, messenger RNA.

The 30 common DEmiRNAs (Co-DEmiRNAs), including 19 consistent DEmiRNAs (6 upregulated and 13 downregulated miRNAs) and 11 inconsistent DEmiRNAs (2 upregulated and 9 downregulated miRNAs from the microarray data and 9 upregulated and 2 downregulated miRNAs from TCGA), were identified through an intersection analysis between the TCGA and microarray datasets (Table 1). In total, 5,479 target mRNAs from 19 consistently Co-DEmiRNAs were identified. Next, 760 Co-DEmRNAs (152 upregulated and 608 downregulated) were identified through the intersection analysis comparing the DEmRNAs in the TCGA and microarray datasets. The miRNA–mRNA regulatory network consists of 19 Co-DEmiRNAs and 760 Co-DEmRNAs (Figure 3). Overall, 19 consistently Co-DEmiRNAs identified 985 targeted relationships with 760 Co-DEmRNAs. Furthermore, the top five miRNAs were identified as hub miRNAs in the network, including miR-539-5p (216 degrees), miR-656-3p (209 degrees), let-7b-5p (135 degrees), miR-2110 (125 degrees), and miR-92b-3p (117 degrees). Finally, 677 Co-DEmRNAs consisted of targeted mRNAs from five hub miRNAs.

**TABLE 1:** Common differentially expressed miRNAs (n = 30) in the GSE151963 and TCGA datasets.

ID	GSE151963			TCGA		
	log2 FC	*p*-value	Expression change	log2 FC	*p*-value	Expression change
**Consistent (n = 19)**						
hsa-miR-369-3p	4.071	2.38E-07	Down	1.434	2.41E-03	Down
hsa-miR-539-5p	3.966	1.06E-07	Down	2.943	6.00E-03	Down
hsa-miR-379-5p	3.727	1.04E-04	Down	1.69	5.53E-03	Down
hsa-miR-494-3p	2.999	7.57E-04	Down	1.837	3.53E-03	Down
hsa-miR-495-3p	2.695	1.06E-03	Down	2.12	4.78E-02	Down
hsa-miR-337-3p	2.634	1.02E-04	Down	2.556	1.69E-04	Down
hsa-miR-376c-3p	2.552	7.87E-06	Down	1.69	3.42E-03	Down
hsa-miR-382-5p	2.51	6.23E-03	Down	1.644	5.36E-03	Down
hsa-miR-154-5p	2.4	1.49E-05	Down	1.837	1.09E-03	Down
hsa-miR-134-5p	2.322	6.94E-03	Down	1.599	1.44E-03	Down
hsa-miR-656-3p	1.914	5.94E-03	Down	1.69	2.61E-02	Down
hsa-miR-655-3p	1.771	8.56E-04	Down	2.184	2.52E-03	Down
hsa-miR-496	1.106	6.55E-04	Down	1.396	3.43E-02	Down
hsa-miR-184	3.775	2.44E-04	Up	2.06	5.86E-11	Up
hsa-miR-486-5p	3.552	5.22E-05	Up	1.8	8.87E-06	Up
hsa-miR-2110	2.483	1.12E-03	Up	1.28	1.40E-09	Up
hsa-miR-92b-3p	1.874	1.41E-06	Up	1.49	8.46E-06	Up
hsa-let-7b-5p	1.806	3.96E-04	Up	1.1	3.26E-08	Up
hsa-miR-455-5p	1.256	4.60E-04	Up	2.566	3.04E-03	Up
**Inconsistent (n = 11)**						
hsa-miR-20a-5p	2.178	3.21E-04	Down	2.63	3.57E-05	Up
hsa-miR-556-5p	2.175	1.85E-04	Down	2.381	1.83E-02	Up
hsa-miR-766-3p	2.089	1.60E-04	Down	1.362	1.48E-02	Up
hsa-miR-146b-5p	1.777	7.07E-05	Down	1.45	2.20E-16	Up
hsa-miR-362-5p	1.661	4.38E-04	Down	1.183	2.23E-03	Up
hsa-miR-130a-3p	1.583	3.90E-05	Down	1.58	2.89E-07	Up
hsa-miR-33a-5p	1.447	8.96E-04	Down	3.38	1.66E-05	Up
hsa-miR-1296-5p	1.439	4.30E-03	Down	1.275	8.47E-03	Up
hsa-miR-552-3p	1.322	1.03E-03	Down	1.49	1.28E-07	Up
hsa-miR-122-5p	4.746	1.14E-05	Up	3.644	3.83E-02	Down
hsa-miR-10b-5p	3.323	3.93E-04	Up	2.06	3.95E-08	Down

*TCGA, Cancer Genome Atlas; FC, fold change.*

### GO and KEGG Analyses of Targeted mRNAs for Hub miRNAs

**FIGURE 4:**
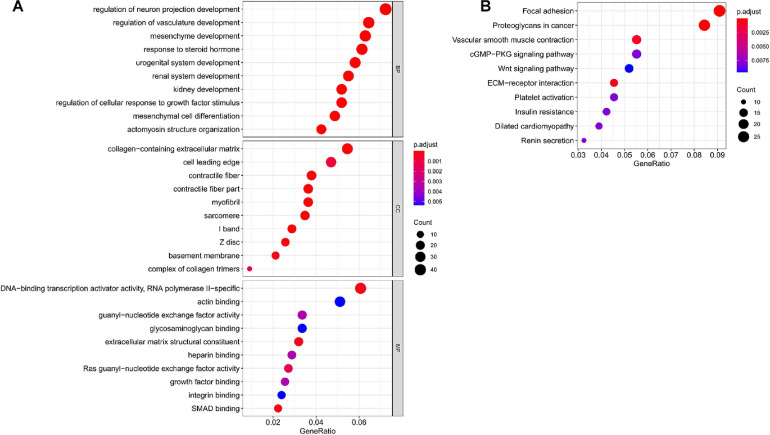
Functional enrichment analysis of targeted mRNAs from the hub miRNAs. **(A)** Dotplot of the top ten results from the GO analysis in terms of BP, CC and MF with the adjusted *p*-value from smallest to largest, respectively, and **(B)** dotplot of the top ten KEGG enrichment pathways. GO, Gene Ontology; BP, biological process; CC, cellular component; MF, molecular function; KEGG, Kyoto Encyclopedia of Genes and Genomes; miRNA, microRNA; mRNA, messenger RNA.

In total, we used 677 Co-DEmRNAs to identify the potential functions of hub miRNAs using GO and KEGG pathway enrichment analyses. We obtained 836 results in the GO analysis, in which Figure 4A illustrates the top 10 results from the biological process, cellular component and molecular function. Some results associated with the occurrence and progression of a tumor, including the regulation of the cellular response to the growth factor stimulus, the collagen-containing extracellular matrix and the basement membrane among others. In addition, 48 signaling pathways emerged from the KEGG pathways analysis (Table 2), with the top ten results shown in Figure 4B. Most of these pathways associated with the occurrence and progression of a tumor, including focal adhesion, proteoglycans in cancer, extracellular matrix–receptor interaction and the Wnt signaling pathway (Table 2). The focal adhesion pathway contained the largest number of mRNAs and the smallest adj. *p*-value.

**TABLE 2:** Results from the Kyoto Encyclopedia of Genes and Genomes enrichment pathway analysis.

ID	Description	*p*-value	Gene ID	Count
hsa04510	Focal adhesion	2.070E-09	PXN/PIK3R3/LAMA4/SPP1/LAMC2/HGF/FYN/AKT3/SHC3/ITGA8/MAPK10/COL4A3/ROCK1/THBS1/IGF1/COL1A1/COL4A2/PPP1R12B/PDGFB/CCND2/PPP1R12C/ITGA5/LAMA2/PRKCB/FLT1/RAPGEF1/CAV2/KDR	28
hsa05205	Proteoglycans in cancer	5.760E-08	WNT7B/PXN/FGFR1/PIK3R3/MMP2/HGF/AKT3/DDX5/ROCK1/WNT3/FZD4/THBS1/IGF1/CDKN1A/COL1A1/PPP1R12B/PPP1R12C/ITGA5/ITPR1/TLR4/WNT7A/PRKCB/TIMP3/HBEGF/CAV2/KDR	26
hsa04512	Extracellular matrix–receptor interaction	5.150E-06	LAMA4/CD36/SPP1/LAMC2/FRAS1/ITGA8/FREM1/COL4A3/THBS1/COL1A1/COL4A2/ITGA5/NPNT/LAMA2	14
hsa04270	Vascular smooth-muscle contraction	1.340E-05	MRVI1/PLA2G4A/GUCY1A2/ROCK1/PLA2G3/EDN1/PPP1R12B/PPP1R12C/ADM/ADCY3/ITPR1/PRKCE/PRKG1/CALCRL/PRKCB/CACNA1C/CACNA1D	17
hsa04022	cGMP–PKG signaling pathway	2.030E-04	MRVI1/PIK3R5/PDE3B/AKT3/GUCY1A2/MEF2A/ROCK1/ADRB2/ADRB1/ADCY3/ITPR1/PRKCE/PRKG1/EDNRB/CACNA1C/PLN/CACNA1D	17
hsa04931	Insulin resistance	2.210E-04	PYGM/PIK3R3/PPP1R3E/PTPN1/CD36/AKT3/PPARGC1A/MAPK10/PPAR GC1B/GFPT2/PRKCE/OGT/PRKCB	13
hsa04611	Platelet activation	2.530E-04	PIK3R3/FCER1G/PIK3R5/PLA2G4A/FYN/AKT3/GUCY1A2/ROCK1/P2RX1/COL1A1/COL3A1/ADCY3/ITPR1/PRKG1	14
hsa04924	Renin secretion	2.590E-04	AQP1/PDE3B/GUCY1A2/ADRB2/EDN1/ADRB1/ITPR1/PTGER4/CACNA1C/CACNA1D	10
hsa05414	Dilated cardiomyopathy	2.700E-04	SGCD/ITGA8/IGF1/ADRB1/ITGA5/ADCY3/ACTC1/CACNB2/LAMA2/CACNA1C/PLN/CACNA1D	12
hsa04310	Wnt signaling pathway	3.810E-04	WNT7B/NKD1/PRICKLE4/SFRP1/RSPO3/SOX17/RSPO2/PRICKLE2/MAPK10/WNT3/FZD4/CCND2/WNT7A/PRKCB/WIF1/AXIN2	16
hsa04933	AGE–RAGE signaling pathway in diabetic complications	3.960E-04	PIK3R3/EGR1/MMP2/AKT3/MAPK10/COL4A3/EDN1/COL1A1/COL4A2/COL3A1/PRKCE/PRKCB	12
hsa04151	PI3K–Akt signaling pathway	4.370E-04	FGFR1/PIK3R3/LAMA4/PIK3R5/SPP1/LAMC2/HGF/AKT3/ITGA8/COL4A3/FOXO3/THBS1/GHR/IGF1/CDKN1A/COL1A1/COL4A2/PDGFB/CCND2/ITGA5/TLR4/ANGPT1/NR4A1/LAMA2/TEK/FLT1/KDR	27
hsa05146	Amebiasis	4.760E-04	PIK3R3/LAMA4/CXCL2/LAMC2/ITGAM/COL4A3/COL1A1/COL4A2/COL3A1/TLR4/LAMA2/PRKCB	12
hsa04360	Axon guidance	5.260E-04	ROBO2/SEMA5A/SEMA6A/PIK3R3/FYN/DPYSL2/ABLIM2/ROCK1/SEMA3D/CFL2/LIMK2/BMPR2/SEMA6D/PLXNA2/SLIT2/SEMA3F/EFNB2	17
hsa04666	Fc gamma R-mediated phagocytosis	7.770E-04	MARCKSL1/PIK3R3/PLA2G4A/AKT3/WASF3/CFL2/GAB2/LIMK2/ASAP1/PRKCE/PRKCB	11
hsa04912	GnRH signaling pathway	7.770E-04	EGR1/MMP2/PLA2G4A/MAPK10/MAP3K3/ADCY3/ITPR1/PRKCB/CACNA1C/HBEGF/CACNA1D	11
hsa01521	EGFR tyrosine kinase inhibitor resistance	7.850E-04	PIK3R3/HGF/AKT3/SHC3/FOXO3/IGF1/PDGFB/NRG1/PRKCB/KDR	10
hsa05418	Fluid shear stress and atherosclerosis	8.150E-04	GSTM5/PIK3R3/MMP2/AKT3/NQO1/MEF2A/MAPK10/DUSP1/EDN1/PDGFB/KLF2/BMPR2/CAV2/KDR	14
hsa04015	Rap1 signaling pathway	1.075E-03	FGFR1/PIK3R3/HGF/AKT3/ITGAM/THBS1/IGF1/PDGFB/ADCY3/DOCK4/ANGPT1/PRKCB/TEK/FLT1/RAPGEF5/RAPGEF1/CNR1/KDR	18
hsa01522	Endocrine resistance	1.206E-03	PIK3R3/MMP2/AKT3/SHC3/MAPK10/IGF1/CDKN1A/NOTCH1/ADCY3/HBEGF/DLL4	11
hsa04072	Phospholipase D signaling pathway	1.504E-03	AGPAT4/PIK3R3/FCER1G/PIK3R5/PLA2G4A/FYN/AKT3/SHC3/DGKH/DNM3/GAB2/PDGFB/ADCY3/MS4A2	14
hsa04935	Growth hormone synthesis, secretion and action	1.875E-03	PIK3R3/AKT3/SHC3/MAPK10/GHR/IGF1/ADCY3/ITPR1/PRKCB/JUNB/CACNA1C/CACNA1D	12
hsa05410	Hypertrophic cardiomyopathy	2.154E-03	SGCD/ITGA8/IGF1/EDN1/ITGA5/ACTC1/CACNB2/LAMA2/CACNA1C/CACNA1D	10
hsa04928	Parathyroid hormone synthesis, secretion and action	2.285E-03	FGFR1/EGR1/AKAP13/MEF2A/PDE4B/CDKN1A/ADCY3/ITPR1/PRKCB/KL/HBEGF	11
hsa05412	Arrhythmogenic right-ventricular cardiomyopathy	2.504E-03	SGCD/DSG2/ITGA8/ITGA5/DSC2/CACNB2/LAMA2/CACNA1C/CACNA1D	9
hsa04929	GnRH secretion	2.817E-03	GABBR1/PIK3R3/SPP1/AKT3/ITPR1/PRKCB/CACNA1C/CACNA1D	8
hsa04066	HIF-1 signaling pathway	2.851E-03	PIK3R3/EGLN3/AKT3/IGF1/EDN1/CDKN1A/TLR4/ANGPT1/PRKCB/TEK/FLT1	11
hsa04014	Ras signaling pathway	3.255E-03	FGFR1/PIK3R3/SYNGAP1/HGF/PLA2G4A/AKT3/SHC3/MAPK10/IGF1/PLA2G3/GAB2/PDGFB/ANGPT1/PRKCB/TEK/FLT1/RAPGEF5/KDR	18
hsa04926	Relaxin signaling pathway	3.686E-03	PIK3R3/MMP2/AKT3/SHC3/MAPK10/COL4A3/EDN1/COL1A1/COL4A2/COL3A1/ADCY3/EDNRB	12
hsa05165	Human papillomavirus infection	3.847E-03	WNT7B/PXN/PIK3R3/LAMA4/SPP1/LAMC2/AKT3/ITGA8/COL4A3/WNT3/FZD4/THBS1/CDKN1A/COL1A1/COL4A2/CCND2/NOTCH1/ITGA5/PTGER4/WNT7A/DLG3/LAMA2/AXIN2	23
hsa05224	Breast cancer	3.960E-03	WNT7B/FGFR1/PIK3R3/AKT3/SHC3/WNT3/FZD4/IGF1/CDKN1A/NOTCH1/WNT7A/AXIN2/DLL4	13
hsa05231	Choline metabolism in cancer	4.036E-03	PIK3R3/PLA2G4A/AKT3/DGKH/WASF3/LYPLA1/MAPK10/PDGFB/GPCPD1/PRKCB	10
hsa04724	Glutamatergic synapse	4.041E-03	SLC1A1/PLA2G4A/DLG4/GRIA1/ADCY3/ITPR1/SLC38A2/SHANK3/PRKCB/CACNA1C/CACNA1D	11
hsa04664	Fc epsilon RI signaling pathway	4.126E-03	PIK3R3/FCER1G/PLA2G4A/FYN/AKT3/MAPK10/GAB2/MS4A2	8
hsa04392	Hippo signaling pathway—multiple species	4.366E-03	DCHS1/FAT4/RASSF2/LIMD1/LATS2	5
hsa04923	Regulation of lipolysis in adipocytes	5.111E-03	PIK3R3/PDE3B/AKT3/ADRB2/ADRB1/ADCY3/PRKG1	7
hsa04071	Sphingolipid signaling pathway	5.598E-03	PIK3R3/FCER1G/SGPP2/FYN/AKT3/MAPK10/ROCK1/GAB2/PRKCE/MS4A2/PRKCB	11
hsa04921	Oxytocin signaling pathway	5.867E-03	PIK3R5/PLA2G4A/GUCY1A2/ROCK1/CDKN1A/PPP1R12B/PPP1R12C/ADCY3/ITPR1/CACNB2/PRKCB/CACNA1C/CACNA1D	13
hsa04910	Insulin signaling pathway	5.979E-03	PYGM/PHKA1/PIK3R3/PPP1R3E/PTPN1/PDE3B/AKT3/SHC3/PPARGC1A/MAPK10/INPP5A/RAPGEF1	12
hsa04115	p53 signaling pathway	6.369E-03	STEAP3/PERP/RRM2/THBS1/IGF1/CDKN1A/MDM4/CCND2	8
hsa05100	Bacterial invasion of epithelial cells	6.369E-03	ARHGEF26/PXN/PIK3R3/SHC3/DNM3/CD2AP/ITGA5/CAV2	8
hsa05202	Transcriptional misregulation in cancer	6.542E-03	TSPAN7/ETV4/NFKBIZ/ETV1/ITGAM/NR4A3/ZEB1/DDX5/SIX4/IGF1/CDKN1A/RUNX1T1/CCND2/LMO2/FLT1	15
hsa04390	Hippo signaling pathway	6.881E-03	WNT7B/NKD1/DLG4/WNT3/FZD4/LIMD1/CCND2/BMPR2/LATS2/SMAD7/WNT7A/DLG3/AXIN2	13
hsa04730	Long-term depression	7.480E-03	PLA2G4A/GUCY1A2/IGF1/GRIA1/ITPR1/PRKG1/PRKCB	7
hsa04024	cAMP signaling pathway	8.340E-03	GABBR1/PIK3R3/PDE3B/AKT3/PDE4B/MAPK10/ROCK1/ADRB2/EDN1/ADRB1/GRIA1/ADCY3/CACNA1C/PLN/HHIP/CACNA1D	16
hsa04550	Signaling pathways regulating pluripotency of stem cells	8.347E-03	WNT7B/FGFR1/PIK3R3/AKT3/WNT3/LIFR/FZD4/IGF1/BMPR2/WNT7A/KLF4/AXIN2	12
hsa00514	Other types of O-glycan biosynthesis	8.432E-03	GALNT15/GALNT7/EOGT/GALNT4/OGT/COLGALT2	6
hsa04010	MAPK signaling pathway	8.579E-03	FGFR1/HGF/PLA2G4A/AKT3/MAP3K8/MAPK10/DUSP1/IGF1/MAP3K3/DUSP7/PDGFB/ANGPT1/NR4A1/CACNB2/PRKCB/TEK/CACNA1C/FLT1/CACNA1D/KDR	20

### Identification of Hub Genes

**FIGURE 5:**
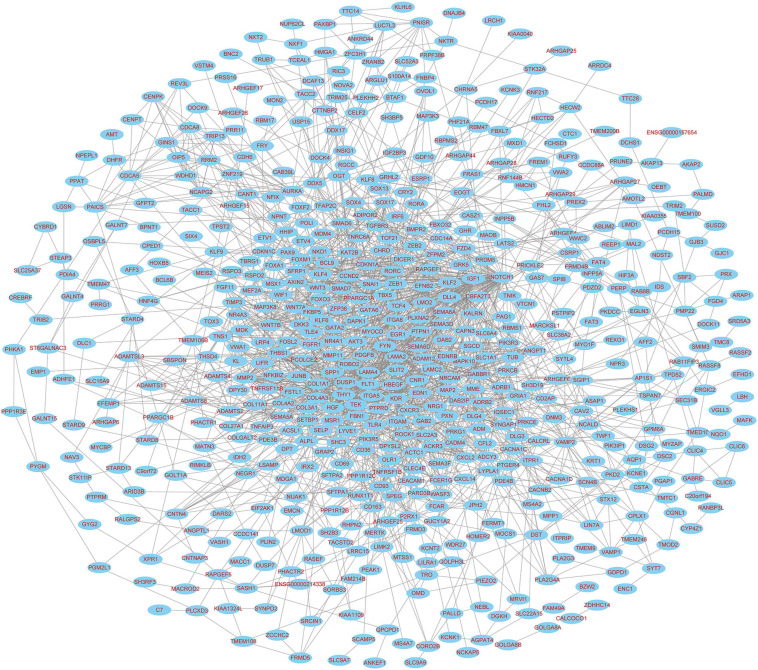
PPI network diagram of targeted mRNAs from the hub miRNAs. PPI, protein–protein interaction; miRNA, microRNA; mRNA, messenger RNA.

**FIGURE 6:**
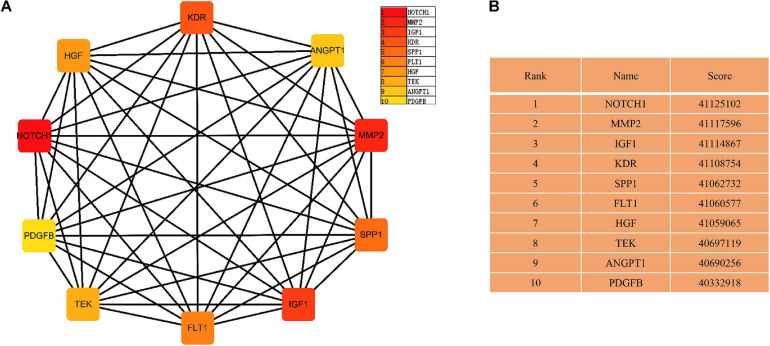
The network of key mRNAs. **(A)** The network diagram of the top ten hub miRNAs and **(B)** the rank and score of the top ten hub mRNAs in the PPI network. PPI, protein–protein interaction; miRNA, microRNA; mRNA, messenger RNA; NOTCH1, neurogenic locus notch homolog protein 1; MMP2, matrix metalloproteinase 2; IGF1, insulin-like growth factor 1; KDR, kinase-insert domain-containing receptor; SPP1, secreted phosphoprotein 1; FLT-1, vascular endothelial growth factor receptor 1; HGF, hepatocyte growth factor; TEK, angiopoietin-1 receptor; ANGPT1, angiopoietin-1 gene; PDGFB, platelet-derived growth factor-beta.

**FIGURE 7:**
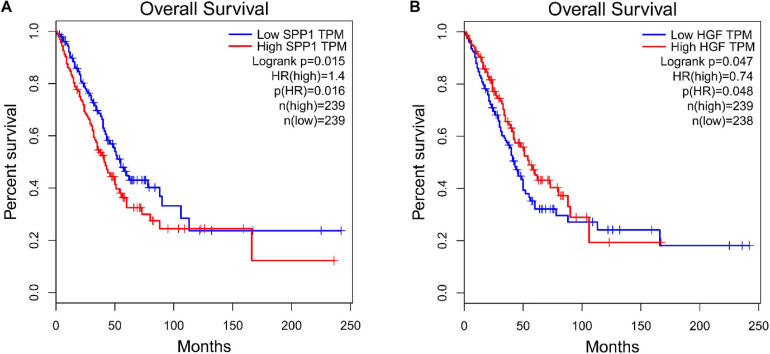
Survival analysis for the hub genes. **(A)** SPP1, secreted phosphoprotein 1 and **(B)** HGF, hepatocyte growth factor. TPF, transcripts per kilobase of exon model per million mapped reads; HR, hazard ratio.

The PPI networks identified 597 mRNAs from 677 Co-DEmRNAs with 2,246 edges related to each other (Figure 5). The top ten key mRNAs were identified using the cytoHubba plugin MCC ranking, including neurogenic locus notch homolog protein 1 (NOTCH1), matrix metalloproteinase 2 (MMP2), insulin-like growth factor 1 (IGF1), kinase-insert domain-containing receptor (KDR), secreted phosphoprotein 1(SPP1), vascular endothelial growth factor receptor 1 (FLT1), hepatocyte growth factor (HGF), angiopoietin-1 receptor (TEK), angiopoietin-1 gene (ANGPT1) and platelet-derived growth factor-beta (PDGFB; Figure 6). These key mRNAs were further verified using GEPIA through the calculation of the survival analysis. Here, only two (SPP1 and HGF) were identified as hub genes and associated with survival (Figure 7). The survival analysis revealed that the expression of SPP1 was negatively correlated with survival, while HGF positively correlated with survival in LUAD (Figure 7). In addition, SPP1 and HGF were both enriched in the focal adhesion signaling pathway (Table 2).

## Discussion

LAUD is a high-risk disease with a high mortality, and its potential occurrence and development mechanisms have not been fully identified. Previously, several studies reported the role of miRNAs in LAUD, although those findings lacked agreement (Cazzoli et al., 2013; Zhong et al., 2018). Here, we identified several hub miRNAs and hub mRNAs in LUAD using the miRNA–mRNA regulatory network using multiple sample types and ethnicities. In total, five hub miRNAs—namely, miR-539-5p, miR-656-3p, let-7b-5p, miR-2110, and miR-92b-3p—played important roles in LUAD tumorigenesis and progression. Two hub mRNAs, namely, SPP1, and HGF, associated with LUAD patient survival. These findings suggest that the specific miRNA and mRNA expression patterns and functional analyses contribute to identifying new predictive markers and therapeutic targets for LUAD patients in clinical settings.

Five hub miRNAs in the network played the most important roles in LUAD tumorigenesis and progression. Specifically, miR-539-5p emerged with the highest node degrees compared to other nodes in the network. Several studies found that miR-539-5p expression levels associated with a poor prognosis and negatively correlated with hypoxia and the stem index among LUAD patients (González-Vallinas et al., 2018; Guo et al., 2020). Yet, further experiments are needed to verify these results given the limited evidence documenting the direct mechanisms of miR-539-5p in LUAD. For example, Chen et al. showed that miR-656-3p could reduce AKT serine/threonine kinase 1 (AKT1) expression and suppress the occurrence and development of non-small cell lung cancer (NSCLC) (Chen et al., 2019). Additionally, upregulating miR-656-3p might improve the chemotherapeutic efficacy in NSCLC by target-regulating sex-determining region Y-related high-mobility group box 4 (SOX4) (Wang et al., 2020). The expression of let-7b-5p was upregulated and might play a vital role in NSCLC based on the GEO dataset using bioinformatics analysis (Zhou et al., 2020), and participate in lymph node micro-metastases of LUAD stage IA (Zhu et al., 2019). miR-2110 and miR-92b-3p express at different levels in different tumors (Long et al., 2017; Gong et al., 2018; Neerincx et al., 2018; Zhao et al., 2018; Ma et al., 2019). To the best of our knowledge, very few studies have attempted to clarify the effect of these two miRNAs in LUAD. In our study, we found that miR-2110 and miR-92b-3p were upregulated in LUAD. Thus, these studies confirmed that miR-539-5p, miR-656-3p, and let-7b-5p are indeed involved in the occurrence and progression of LUAD. Furthermore, miR-92b-3p and miR-2110 may represent new potential therapeutic targets in LUAD.

SPP1 and HGF were identified as hub mRNAs in PPI and significantly predicted survival outcomes in LUAD. These two mRNAs appear enriched in the focal adhesion signaling pathway, containing the largest number of mRNAs with the smallest significant adj. *p*-value. The focal adhesion signaling pathway could involve the tumor microenvironment (TME), resulting in tumor progression and indicative of a poor outcome in LUAD (Yue et al., 2019; Wei et al., 2020). Accumulating evidence has revealed that the TME could influence malignant behavior and the progression of tumors. Previous studies found that SPP1 played a role in lung cancer escape and mediating macrophage polarization, while inhibiting SPP1 expression might overcome resistance to second-generation epidermal growth factor receptor gene (EGFR)–tyrosine kinase inhibitors (TKIs) (Zhang et al., 2017; Wang et al., 2019). HGF is a pleiotropic cytokine composed of an α-chain and a β-chain, which mediates malignant biological behaviors in LUAD, including growth, invasion, metastasis and the epithelial-to-mesenchymal transition (EMT), as well as increases resistance to EGFR–TKIs in LUAD (Tarhini et al., 2017; Suzuki et al., 2019). Importantly, it is worth noting that SPP1 presented with the targets of miR-539-5p, correlated with a poor survival in LUAD and appeared involved in the regulation of focal adhesion and the extracellular matrix–receptor interaction pathways. Thus, we found a significant miR-539-5p–SPP1 axis, which played important roles in LUAD occurrence and progression by regulating the TME through the aforementioned pathways in our regulatory network. Although the other eight key mRNA played some important roles in LUAD or lung cancer (Ding et al., 2008; Roybal et al., 2011; Licciulli et al., 2013; Li et al., 2017; Neri et al., 2017; Tian et al., 2018; Yao et al., 2019), these were not the significant predictors of survival in LUAD.

Aside from the strengths of our study, we should point out several limitations. Firstly, the small sample size in our cohort mirrors the limitations of similar previous studies (Patnaik et al., 2012; Xu et al., 2020). Yet, all of these miRNA expression profiling studies offer opportunities to develop new therapeutic targets. Although increasing numbers of miRNAs have been identified, a meta-analysis approach and a larger cohort are needed in future in order to minimize the drawbacks resulting from small sample sizes and different technological platforms. Secondly, given our findings related to the target genes from the Co-DEmiRNAs and hub miRNAs identified primarily based on bioinformatics analyses, further mechanistic studies from cells as well as animal models and clinical validation studies may be necessary in the future.

In this study, we constructed a miRNA–mRNA regulatory network for LUAD, identifying five hub miRNAs (namely, miR-539-5p, miR-656-3p, let-7b-5p, miR-2110, and miR-92b-3p) and two hub mRNAs (SPP1and HGF) through our microarray data and TCGA dataset. We also performed a functional enrichment analysis on these final target genes to understand the potential functional mechanisms in LUAD. These findings provide new molecular markers for the prediction, prognosis and therapeutic targets in clinical settings, as well as emphasize new mechanistic insights into LUAD.

## Data Availability Statement

The datasets presented in this study can be found in online repositories. The names of the repository/repositories and accession number(s) can be found in the article/Supplementary Material.

## Ethics Statement

The studies involving human participants were reviewed and approved by the Ethics Committee of the Gansu Provincial Hospital, China. The patients/participants provided their written informed consent to participate in this study.

## Author Contributions

QY, X-JW, and JG contributed to the design of the study and revised the manuscript. X-JW and ZW performed the sample collection, analysis and downloaded the data. X-JW and JG contributed to the data analysis and writing the manuscript. All authors approved the final version of the manuscript.

## Conflict of Interest

The authors declare that the research was conducted in the absence of any commercial or financial relationships that could be construed as a potential conflict of interest.

## Footnotes

1 http://gdac.broadinstitute.org/

2 http://www.mirdb.org/

3 http://www.targetscan.org/

4 zmf.umm.uni-heidelberg.de/mirwalk2

5 http://string-db.org

6 http://gepia.cancer-pku.cn

## Supplementary Material

Supplementary Table S1 Clinical characteristics of LUAD patients in the microarray data (GSE151963).

Supplementary Table S2 Clinical characteristics of LUAD patients in the TCGA data.
