# Supplementary material for: Identification of a Potentially Functional microRNA–mRNA Regulatory Network in Lung Adenocarcinoma Using a Bioinformatics Analysis
Source: Front Cell Dev Biol. 2021 Feb 18;9:641840. doi: 10.3389/fcell.2021.641840 (PMC7930498; doi:10.3389/fcell.2021.641840)
Supplement: Supplementary Table S1 — Clinical characteristics of LUAD patients in the microarray data (GSE151963). [file Table_1.DOCX]

Supplementary Material

# Supplementary Tables

**Table S1.** Clinical characteristics of LUAD patients in microarray data (GSE151963).

| **Case** | **Gender** | **Age (year)** | **Ethnicity** | **AJCC stage** | **Outcome** |
| --- | --- | --- | --- | --- | --- |
| 1 | Female | 57 | Asian | Stage IV | Alive |
| 2 | Male | 66 | Asian | Stage III | Dead |
| 3 | Male | 54 | Asian | Stage III | Alive |
| 4 | Female | 61 | Asian | Stage IV | Dead |
| 5 | Male | 74 | Asian | Stage III | Dead |
| 6 | Female | 64 | Asian | Stage IV | Dead |

Note: LUAD, Lung adenocarcinoma; AJCC, American Joint Committee on Cancer.
