# Supplementary material for: Identification of a Potentially Functional microRNA–mRNA Regulatory Network in Lung Adenocarcinoma Using a Bioinformatics Analysis
Source: Front Cell Dev Biol. 2021 Feb 18;9:641840. doi: 10.3389/fcell.2021.641840 (PMC7930498; doi:10.3389/fcell.2021.641840)
Supplement: Supplementary Table S2 — Clinical characteristics of LUAD patients in the TCGA data. [file Table_2.DOCX]

Supplementary Material

# Supplementary Tables

**Table S2.** Clinical characteristics of LUAD patients in TCGA data.

| Clinical factors | Number of patients |
| --- | --- |
| **Gender** |  |
| Male (n, %) | 238 (46.2%) |
| Female (n, %) | 277 (53.8%) |
| **Age, in years** (mean, 66 years old) |  |
| <66 (n, %) | 246 (47.8%) |
| ≥66 (n, %) | 250 (48.5%) |
| Not available | 19 (3.7%) |
| **Ethnicity** |  |
| Black or African American (n, %) | 52 (10.1%) |
| White (n, %) | 388 (75.3%) |
| American Indian or Alaskan native (n, %) | 1 (0.2%) |
| Asian (n, %) | 8 (1.6%) |
| Not available (n, %) | 66 (12.8%) |
| **AJCC stage** |  |
| Stage I (n, %) | 275 (53.4%) |
| Stage II (n, %) | 122 (23.7%) |
| Stage III (n, %) | 84 (16.3%) |
| Stage IV (n, %) | 26 (5.0%) |
| Discrepancy (n, %) | 7 (1.4%) |
| Not available (n, %) | 1 (0.2%) |

Note: LUAD, Lung adenocarcinoma; AJCC, American Joint Committee on Cancer; TCGA, the Cancer Genome Atlas.
